# Protein expression of nucleolar protein 12 in the retina and its implication in protection of retina from UV irradiation damage

**DOI:** 10.1038/s41420-024-01902-x

**Published:** 2024-03-11

**Authors:** Jingtao Liu, Xiaomei Tan, Li Li, Liying Cao, Yan Zhou, He Li, Ting Peng

**Affiliations:** 1 Department of Histology and Embryology, School of Basic Medical Sciences, Tongji Medical College, Huazhong University of Science and Technology, Wuhan, 430030 People’s Republic of China; 2 Department of Histology and Embryology, Hubei University of Medicine, Shiyan, 442000 People’s Republic of China; 3 Department of Critical Care Medicine, Zhongnan Hospital of Wuhan University, Wuhan, 430071 People’s Republic of China; 4 Department of Histology and Embryology, School of Medicine, Yunnan University, Kunming, 650091 People’s Republic of China

**Keywords:** Retina, Cell death in the nervous system

## Abstract

Nucleolar protein 12 (NOL12), one of the nucleolar proteins which are primarily expressed in the nucleolus and play key roles in RNA metabolism, cell proliferation, cell cycle, and cell survival, is widely expressed in various species and multiple organs. Although it has been reported that the mRNA of *Drosophila* NOL12 homolog viriato is expressed in the eyes of *Drosophila*, the protein expression of NOL12 in mammalian eyes remains to be elucidated. In this study, we showed through immunohistochemistry that NOL12 was present in the rat retina, with predominant distribution in the cytoplasm of the retinal neuronal cells. In the human retinoblastoma cell line WERI-Rb1, we found that altering NOL12 expression led to a change in WERI-Rb1 cell viability. Knocking down NOL12 expression decreased cell viability. In contrast, overexpressing NOL12 increased cell viability. Furthermore, increasing NOL12 expression inhibited ultraviolet (UV)-induced apoptosis. These findings demonstrated that NOL12 may play an important protective role in retinal cells. In the WERI-Rb1 cells exposed to UV irradiation, we detected that NOL12 was degraded, but this degradation could be attenuated by a pan-Caspase inhibitor. Notably, the inhibitory effect of NOL12 against UV-induced apoptosis could be restrained by increasing the expression of ATR serine/threonine kinase (ATR), a kinase that, when activated by severe DNA damage, can result in apoptosis. We also found that upregulating NOL12 inhibited the activation of ATR caused by UV irradiation. Additionally, inhibiting ATR activity reduced apoptosis resulting from both silencing NOL12 expression and UV exposure. Thus, NOL12 may protect against UV irradiation-induced retinal damage by inhibiting ATR activity.

## Introduction

Nucleolar proteins are a group of proteins which are predominantly expressed in the nucleolus and play regulatory roles in RNA metabolism, cell cycle, DNA damage response (DDR), and apoptosis [1–4]. Nucleolar protein 12 (NOL12), originally named 25 kDa nucleolar protein (Nop25), is widely expressed in various species and multiple organs [5]. Northern blotting or RT-PCR analysis has detected the ubiquitous expression of Nop25 mRNA in the brain, lung, spleen, liver, kidney, heart, and testis of mice [5], and immunostaining has demonstrated expression of NOL12 protein in the nucleoli of human HCT116 cells, HeLa cells, primary dermal fibroblasts, and COS-7 cells [6–8]. Moreover, the mRNA of *Drosophila* NOL12 homolog viriato has been found in the *Drosophila* eyes [9, 10]. However, in the mammalian eyes, much is unknown about the expression of NOL12 at the protein level.

Like other nucleolar proteins, NOL12 plays an essential role in the regulation of RNA metabolism and maintenance of nucleolar integrity [6–8, 11]. It has also been found that NOL12-mediated nucleolar stress negatively impacts both the cell proliferation and cell cycle [6, 7], with the inhibition of the cell cycle by NOL12 being p53 dependent [7]. In addition, NOL12 plays a critical role in cell survival [6, 9]. In the eyes of *Drosophila*, viriato knockdown with RNAi resulted in increased Caspase-mediated apoptosis of the cells in the anterior domain of the eye disc [9]. In the HCT116 cells, loss of NOL12 led to the upregulation of DNA damage marker γ-H2AX expression, impaired recovery of cells from DNA stress, and increased apoptotic response [6]. Notably, the apoptotic response triggered by the silencing of NOL12 in HCT116 cells could not be blocked by the repression of p53 but could be mitigated by inhibiting ATR serine/threonine kinase (ATR) [6], which can be activated by DNA damage stress, especially by DNA breaks induced by ultraviolet (UV) irradiation [12, 13].

ATR is a central member of the family of phosphatidylinositol-3 kinase-related kinases (PIKKs) [14]. Upon activation, ATR phosphorylates the checkpoint kinase 1 (Chk1) and SMARCAL1, which cause cell cycle arrest, and repair of damaged DNA resulting from stress [12, 13]. However, when the DNA damage stress is severe, activated ATR phosphorylates checkpoint kinase 2 (Chk2) or p38-mitogen activated protein kinase (MAPK). This leads to the inability to repair DNA damage, ultimately resulting in cell apoptosis [15, 16]. Exposure to UV irradiation causes severe DNA damage in the organs, including the eyes [17–19]. In retinal pigment epithelial cells, exposure to UV irradiation significantly increased the formation of DNA tails, indicating substantial DNA damage as detected by the comet assay [20]. In the pupal retina of *Drosophila*, UV irradiation rapidly induced pyrimidine dimers, causing DNA damage that ultimately resulted in retinal morphology aberrations and cell apoptosis [21]. Given the expression of viriato in Drosophila eyes, the vital role of NOL12 in cell survival, and the suppression of ATR inhibitor on NOL12 deficiency-induced apoptosis [6, 9, 10], NOL12 is suggested to play a protective role in the eyes exposed to UV irradiation by inhibiting ATR.

In this study, we demonstrate that NOL12 is expressed in the retinal nerve layer of the rat eye and plays a protective role against UV irradiation injury in retinal cells. We find that UV irradiation induces Caspase-dependent degradation of NOL12 in the retinal cells. In addition, we show that overexpression of NOL12 decreases ATR activation and apoptosis in the UV-irradiated retinal cells, and that ATR inhibition alleviates damage of retinal cells caused by downregulation of NOL12 expression or UV irradiation.

## Results

### NOL12 is expressed in the retinal neuronal cells of the rat eyes

**Fig. 1:**
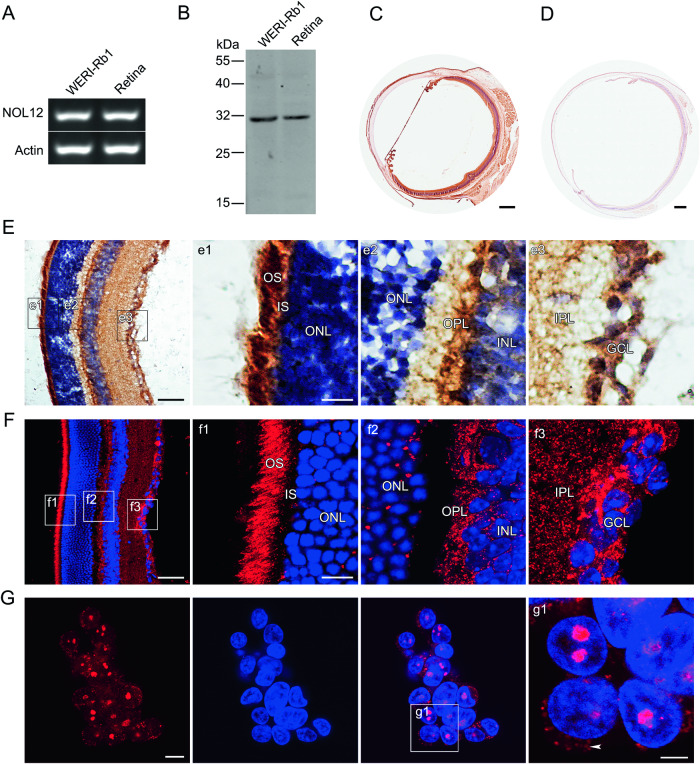
NOL12 is mainly expressed in the retinal neuronal cells of the rat eyes. **A**, **B** Expression analyses of NOL12 in adult rat eyes and WERI-Rb1 cells by RT-PCR (**A**) and Western blot (**B**). **C**, **D** Expression analyses of NOL12 in the adult rat eyes by ABC method of immunohistochemical staining (ABC staining) (**C**) and the absorption experiment (**D**). **E**–**G** Immunohistochemical examination of NOL12 distribution in the retinas of adult rats (**E**, **F**) and WERI-Rb1 cells (**G**) by ABC staining (**E**) and immunofluorescence staining (**F**, **G**); high-power images of the boxed areas in **E**–**G** are shown in e1-e3, f1-f3, and g1. NOL12 predominantly localizes in the cytoplasm of cells in the retinal nerve layer of adult rats (**E**, **F**) but in the nucleus of WERI-Rb1 cells (**G**); arrowheads indicate the low levels of cytoplasmic localization of NOL12 in WERI-Rb1 cells (g1). A rabbit polyclonal antibody against NOL12 was used in Western blotting (**B**) and immunohistochemical staining, including ABC staining (**C**–**E**) and immunofluorescence staining (**F**, **G**); NOL12 was labeled by RRX (red), and nuclei were counterstained with Hoechst 33258 (blue). GCL ganglion cell layer, IPL inner plexiform layer, INL inner nuclear layer, OPL outer plexiform layer, ONL outer nuclear layer, IS inner segments, and OS outer segments. Scale bar: 500 μm in (**C**, **D**), 50 μm in (**E**, **F**), 20 μm in (e1, f1), 10 μm in (**G**), and 5 μm in (g1).

In order to determine whether NOL12 is expressed in the rat eye, the total RNA and protein were extracted from the eyeballs of the adult rats, and subjected to RT-PCR and Western blot analyses. High levels of NOL12 transcript, as well as its translation product NOL12 protein with a molecular weight of 32 kDa, were detected in the eyeballs (Fig. 1A, B). To verify the distributive pattern of NOL12 in the eye, we carried out immunohistochemical staining on the rat eyeball sections. The NOL12 protein was demonstrated to be primarily expressed in the retina, with positive staining in the retinal nerve layer and negative staining in retinal pigment epithelial cells (Fig. 1C, D and Supplementary Fig. S1). In the retinal nerve layer, NOL12 immunoreactive products were found to be located mainly in the retinal ganglion cells (RGCs) layer, the outer segments of photoreceptors, and the inner and outer plexiform layers (Fig. 1E, F). This localization pattern indicates that NOL12 is primarily expressed in the cytoplasm of cells in the retinal nerve layer of adult rats.

The expression of NOL12 in the WERI-Rb1 cell, a human retinoblastoma cell line [22, 23], was also examined using RT-PCR, Western blotting, and immunofluorescent staining. The mRNA of NOL12 of approximate length was detected in WERI-Rb1 cells, similar to that observed in rat eyes (Fig. 1A). In addition, the presence of NOL12 protein in WERI-Rb1 cells was confirmed using the same antibody employed for detecting NOL12 protein in the rat eyes (Fig. 1B, G). We noted that the molecular weight of the NOL12 protein, as determined by Western blotting, was slightly lower in WERI-Rb1 cells than in the rat eyes (Fig. 1B). Furthermore, immunofluorescent staining revealed that the NOL12 protein was primarily localized in the nucleus of WERI-Rb1 cells, with a low level of cytoplasmic expression (Fig. 1G).

**Fig. 2:**
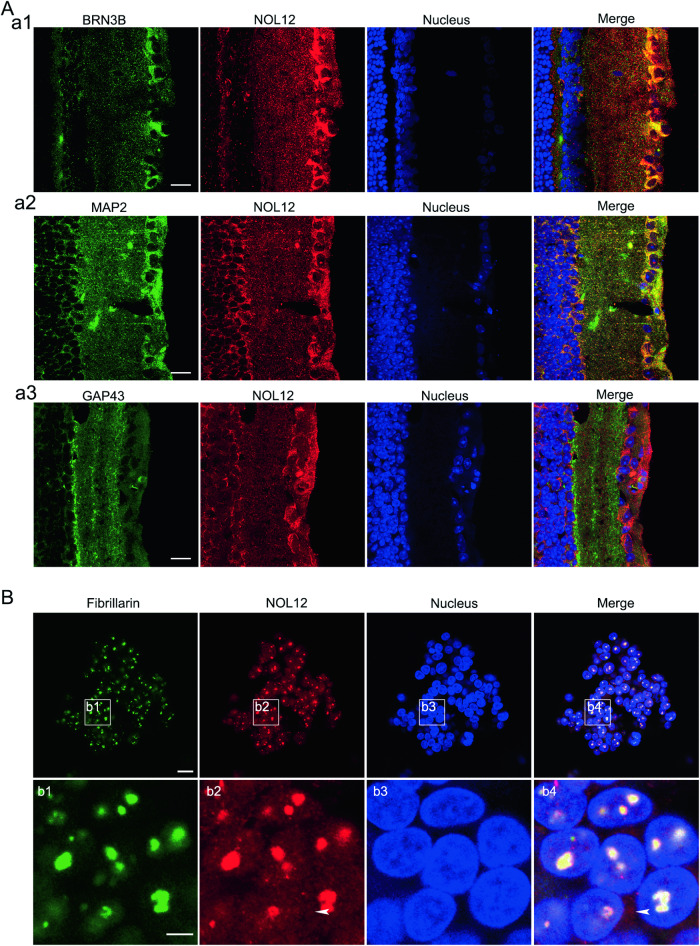
Differential subcellular localization of NOL12 in the rat retinas and WERI-Rb1 cells. **A** Double immunofluorescence staining for analyzing subcellular localization of NOL12 in the cells of rat retinal tissue, showing the colocalization of NOL12 with the RGCs marker BRN3B (a1) and the dendrite marker MAP2 (a2), but not with the axonal terminal marker GAP43 (a3). **B** Double immunofluorescence staining of NOL12 and the nucleolar marker fibrillarin in WERI-Rb1 cells showing that NOL12 is primarily distributed in the nucleolus, with a low level of cytoplasmic distribution (arrowheads). High-power images of the boxed areas in **B** are shown in b1–b4. A rabbit polyclonal antibody against NOL12 was used in immunofluorescence staining in a1, a3, and **B**, while a mouse monoclonal antibody against NOL12 was used in immunofluorescence staining in a2. NOL12 was labeled by RRX (red), BRN3B, GAP43, MAP2, and fibrillarin were labeled by FITC (green), and nuclei were counterstained with Hoechst 33258 (blue). RGCs, retinal ganglion cells. Scale bar: 20 μm in **A** and **B**, 5 μm in b1.

To further clarify the subcellular localization of NOL12 within retinal neurons, we performed double immunofluorescent labeling. NOL12 was labeled alongside one of several neuron markers: POU class 4 homeobox 2 (BRN3B) for ganglionic cells, growth associated protein 43 (GAP43) for axon terminals, and microtubule associate protein 2 (MAP2) for dendrites. We found that NOL12 predominantly localized to the cytoplasm of retinal cells, showing minimal, if any, presence in the nucleus (Fig. 2A). In ganglionic cells, we observed a strong colocalization of NOL12 with BRN3B in the perikaryon (Fig. 2a1). Conversely, in the inner plexiform layers, NOL12 localization was exclusive to MAP2-labeled dendrites (Fig. 2a2), not GAP43-labeled axon terminals (Fig. 2a3).

Additional double immunofluorescence staining of NOL12 along with the nucleolar marker fibrillarin revealed that NOL12 was primarily localized in the nucleolus of WERI-Rb1 cells, although a low level of cytoplasmic localization was also observed (Fig. 2B). To clarify whether the differences in NOL12 localization in the retina of adult rats versus WERI-Rb1 cells are attributable to the developmental differentiation of retinal cells, we compared the NOL12 localization in rat retinal cells at different stages of development. Immunofluorescence staining showed that at embryonic day 13.5 (E13.5), NOL12 was abundantly expressed in the nuclei with less expression in the cytoplasm of the inner neuroblastic layer (INBL); however, by postnatal day 1 (P1) and in adulthood (6–8 weeks old), it was mainly observed in the cytoplasm with reduced expression in the nuclei of the ganglion cells (Supplementary Fig. S1).

### NOL12 protects WERI-Rb1 cells against UV irradiation-induced injury

**Fig. 3:**
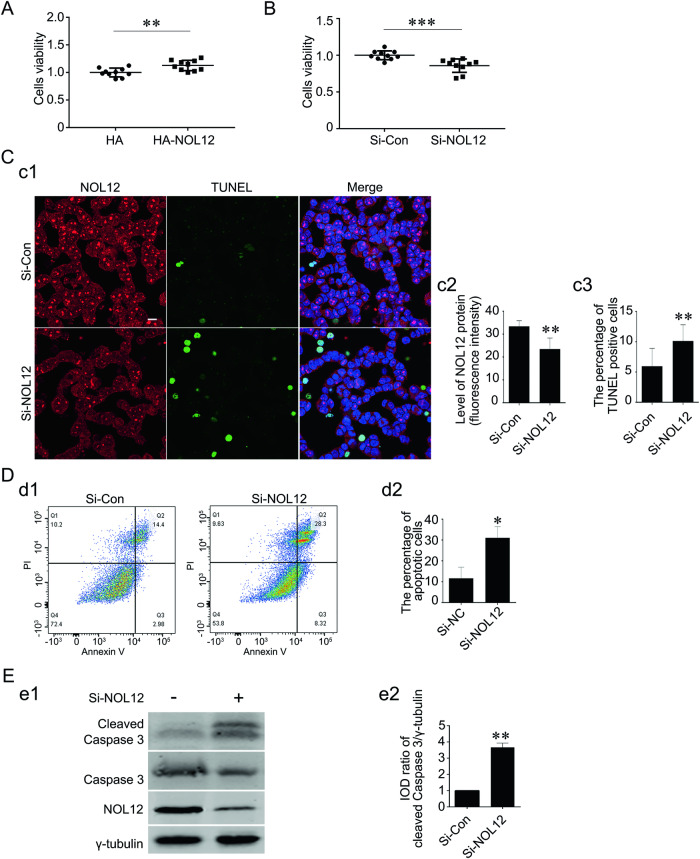
Inhibition of NOL12 expression leads to the apoptosis of retinal cells. **A**, **B** The effect of overexpression of NOL12 (**A**) and NOL12 silence (**B**) on the viability of the WERI-Rb1 cell was examined by Alamar blue analysis. **C** The effect of silencing NOL12 expression with Si-NOL12 on apoptosis in WERI-Rb1 cells was analyzed by TUNEL staining. A rabbit polyclonal antibody against NOL12 was used to stain NOL12 (labeled with RRX [red]), a TUNEL staining kit labeled apoptotic cells (green), and Hoechst 33258 (blue) counterstained the nuclei (c1). The protein level of NOL12 detected by immunofluorescence staining (c2) and the apoptotic rate measured by TUNEL assay (c3) were statistically analyzed. **D** Flow cytometric analysis of the effect of NOL12 expression inhibition by Si-NOL12 on apoptosis in WERI-Rb1 cells: Annexin V/PI flow cytometry was utilized to determine the apoptotic cells (d1), and the percentage of apoptotic cells was statistically analyzed (d2). **E** The effect of NOL12 silence on the expression of cleaved Caspase-3 was detected by Western blot analysis using a rabbit polyclonal antibody against NOL12. Representative Western blot of cleaved Caspase-3, Caspase-3, and NOL12 are shown (e1), and the band intensity of cleaved Caspase-3 was analyzed (e2). Data were expressed as mean ± SD. *n* = 10 for **A** and **B**, and *n* = 3 for **C**–**E**; *P < 0.05, **P < 0.01. Scale bar, 10 μm.

To determine whether NOL12 expressed in retinal cells plays a protective role, we first analyzed the effects of altering NOL12 expression levels on the viability and apoptosis of WERI-Rb1 cells. The Alamar blue assay indicated that overexpression of NOL12 enhanced the viability of WERI-Rb1 cells, whereas silencing NOL12 expression reduced it (Fig. 3A, B). In WERI-Rb1 cells with NOL12 knockdown by Si-NOL12, a significantly higher number of apoptotic cells were detected compared to control cells, as evidenced by both TUNEL staining and flow cytometry using annexin V-PI labeling (Fig. 3C, D). Furthermore, Western blot analysis revealed that treatment with Si-NOL12 led to a significant increase in cleaved Caspase-3 levels, compared to those in WERI-Rb1 cells treated with Si-Con (Fig. 3E).

**Fig. 4:**
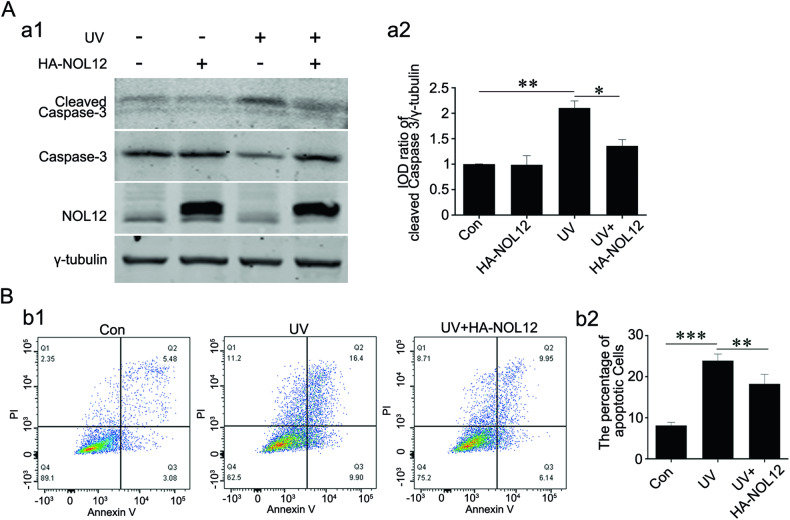
NOL12 protects retinal cells from UV irradiation-induced apoptosis. **A** Western blotting was employed to assess the impact of NOL12 overexpression on the expression of cleaved Caspase-3 in WERI-Rb1 cells under UV irradiation. A rabbit polyclonal antibody against NOL12 was used to detect the expression of NOL12 (a1). Representative Western blot of cleaved Caspase-3, Caspase-3, and NOL12 are shown (a1), and the band intensity of cleaved Caspase-3 was analyzed (a2). **B** Flow cytometry analysis representing the inhibitory effect of NOL12 overexpression on apoptosis in WERI-Rb1 cells under UV irradiation. Apoptotic cells were determined by Annexin V/PI flow cytometry (b1), and the percentages of apoptotic cells was statistically analyzed (b2). Data were expressed as mean ± SD. *n* = 3. *P < 0.05; **P < 0.01.

The retina is highly sensitive and prone to damage from UV irradiation, which can induce apoptotic lesions [24, 25]. To further investigate the protective role of NOL12 in the retina, we examined the effect of NOL12 overexpression on UV exposure-induced apoptosis in the WERI-Rb1 cells. Western blotting and flow cytometric analysis revealed that the marked increase in cleaved Caspase-3 level and apoptotic cell percentage caused by UV irradiation in the WERI-Rb1 cells could be restrained by overexpression of NOL12 (Fig. 4A, B). This demonstrated that NOL12 can protect retinal cells from UV irradiation-induced damage, including cell apoptosis.

### UV irradiation leads to a Caspase-dependent degradation of NOL12

**Fig. 5:**
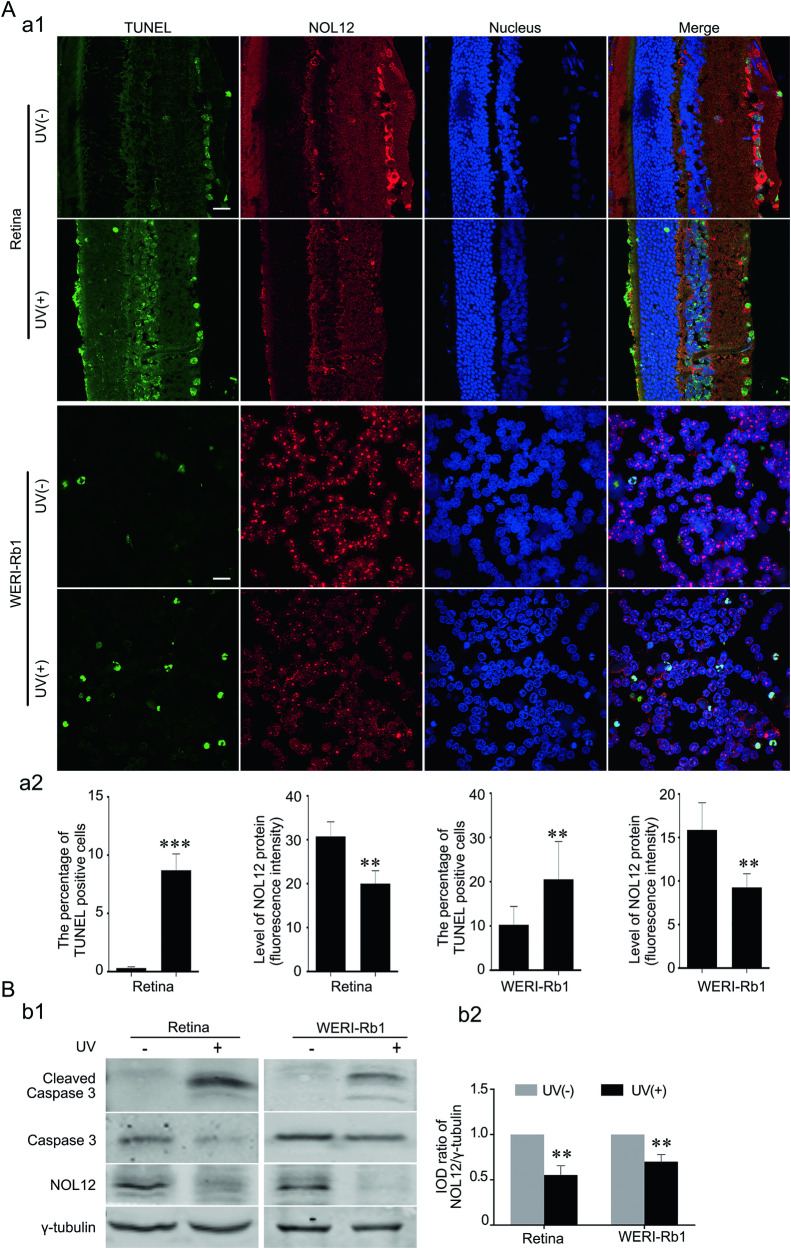
UV irradiation induces retina injury accompanied by the decrease of NOL12 protein. **A** The immunofluorescence and TUNEL double staining revealed apoptosis induced by UV, accompanied by a decrease in NOL12 protein levels in UV-irradiated retinas and WERI-Rb1 cells. A rabbit polyclonal antibody against NOL12 was used to stain NOL12 (labeled with RRX [red]), a TUNEL staining kit labeled apoptotic cells (green), and Hoechst 33258 (blue) counterstained the nuclei (a1); the protein level of NOL12 detected by immunofluorescence staining and the apoptotic rate measured by TUNEL assay were statistically analyzed (a2). **B** The effect of UV on the expression of cleaved Caspase-3 was detected by Western blot analysis using a rabbit polyclonal antibody against NOL12. Representative Western blots of cleaved Caspase-3, Caspase-3, and NOL12 are shown (b1), and the band intensity of NOL12 was analyzed (b2). Data were expressed as mean ± SD. *n* = 3. *P < 0.05; **P < 0.01; ***P < 0.001. Scale bar, 20 μm.

**Fig. 6:**
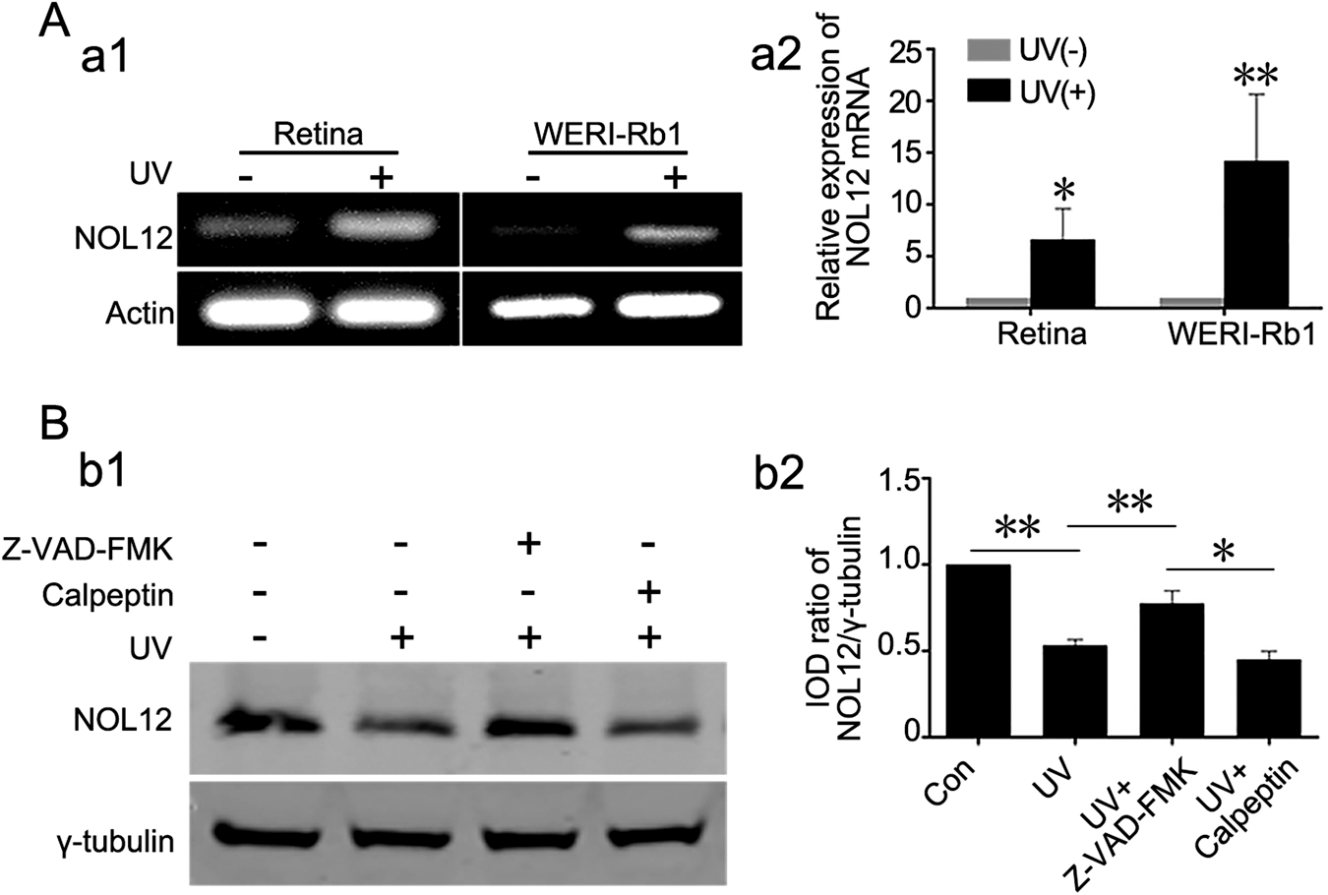
UV irradiation degrades NOL12 through the Caspase activation-dependent manner. **A** Analysis of NOL12 mRNA expression in rat retinas and WERI-Rb1 cells following UV irradiation was conducted using semi-quantitative RT-PCR (a1) and qRT-PCR (a2). **B** Detection of NOL12 protein expression by Western blotting demonstrates that Z-VAD-FMK inhibits the UV-induced decrease in NOL12 protein level, while Calpeptin does not. Rabbit polyclonal antibody against NOL12 was used in Western blot. A representative Western blot of NOL12 is shown (b1), and the band intensity was analyzed (b2). Z-VAD-FMK, the inhibitor of pan-Caspase kinases. Calpeptin, the inhibitor of pan-calpain kinases. Data were expressed as mean ± SD. *n* = 3. *P < 0.05; **P < 0.01.

In the aforementioned experiment, where Western blotting was used to analyze the effect of NOL12 overexpression on UV-induced apoptosis, it was found that UV irradiation not only markedly increased the level of activated Caspase-3 but also significantly decreased the level of endogenous NOL12 (Fig. 4A). Further investigations consistently revealed that, in the retina of the rat and WERI-Rb1 cells exposed to UV radiation, a noticeable decline in NOL12 immunoreactivity was observed alongside significant increase in both the number of TUNEL-positive cells and the activated Caspase-3 level (Fig. 5). These results, showing UV radiation-induced cell apoptosis accompanied by a reduction in endogenous NOL12 protein levels in the retina or retinal cells, prompted us to investigate whether UV radiation could cause inhibition of NOL12 expression. To this end, we examined the effect of UV irradiation on the expression of NOL12 mRNA in the rat retina and in human retinal cells WERI-Rb1 using semi-quantitative RT-PCR and qRT-PCR analyses. Surprisingly, the results indicated that UV irradiation did not inhibit the expression of NOL12 mRNA; instead, it up-regulated it (Fig. 6A), suggesting that UV irradiation might provoke the degradation of NOL12 protein in retinal tissue or cells.

Therapeutic proteins in the retina, such as *Drosophila* inhibitor of apoptosis protein-1 (Diap1) and sirtuin-1 (SIRT1), are known to be sensitive to degradation by UV irradiation [21, 26, 27]. Concurrently, UV irradiation can activate various proteases, including Caspases, which contribute to the degradation of the therapeutic proteins [28]. Utilizing SitePrediction (https://www.dmbr.ugent.be/prx/bioit2-public/ SitePrediction/), we identified potential cleavage sites for Caspases and calpains on NOL12 molecule (Supplementary Table S2). Subsequently, we performed Western blotting to examine the role of these two proteases in the UV-induced degradation of NOL12 in retinal cells. Our findings revealed that the pan-Caspase inhibitor Z-VAD-FMK significantly attenuated the reduction of NOL12 protein levels in WERI-Rb1 cells exposed to UV light, whereas the pan-calpain inhibitor calpeptin did not have a similar effect (Fig. 6B). These results therefore confirm that UV irradiation can lead to the degradation of NOL12 in retinal tissue or cells through the activation of Caspases.

### NOL12 alleviates retinal apoptosis and activation of ATR induced by UV

**Fig. 7:**
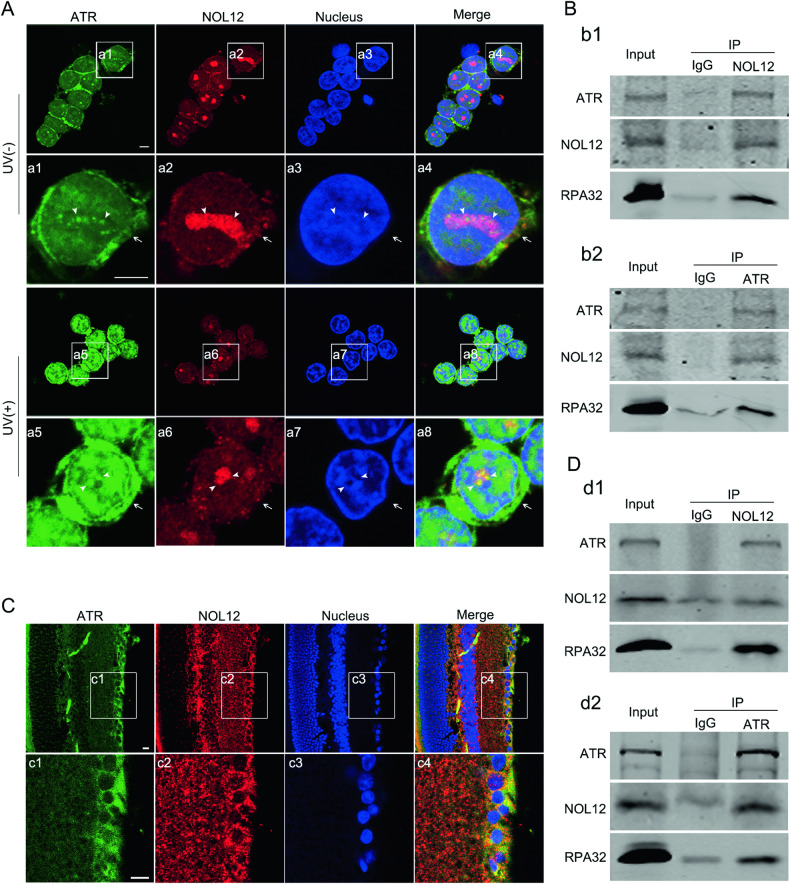
NOL12 interacts with ATR in the rat retina and WERI-Rb1 cells. **A** Double immunofluorescence staining of NOL12 and ATR in WERI-Rb1 cells showing that NOL12 is well colocalized with ATR in WERI-Rb1 cells, predominantly in the nucleoli (arrowheads) and to a lesser extent in the cytoplasm (arrows). High-power images of the boxed areas in (**A**) are shown in a1–a8. **B** The interactions between NOL12, ATR, and its substrate RPA in WERI-Rb1 cells were detected by co-immunoprecipitation assays. **C** Double immunofluorescence staining of NOL12 and ATR in adult rat retinas showing that NOL12 is well colocalized with ATR in the cytoplasm of RGCs. High-power images of the boxed areas in (**C**) are shown in c1–c4. **D** The interactions between NOL12, ATR, and its substrate RPA in adult rat retinas were detected by co-immunoprecipitation assays. Rabbit polyclonal antibody against NOL12 and mouse monoclonal antibody against ATR was utilized in immunofluorescence staining (**A**, **C**). Rabbit polyclonal antibodies against NOL12 and ATR were utilized in Western blot (**B**, **D**). NOL12 is labeled by RRX (red), ATR by FITC (green), and nuclei are counterstained with Hoechst 33258 (blue) (**A**, **C**). Scale bars: 5 μm in **A** and 10 μm in **C**.

Given that UV-induced apoptosis is associated with the activation of ATR [16, 19, 25], and the HCT116 cell apoptosis caused by silencing NOL12 can be blocked by ATR inhibitor, rather than the inhibitor of another PIKKs family member ATM serine/threonine kinase (ATM) [6], it is therefore hinted at that changes in NOL12 expression may affect the ATR activity. To address how the UV irradiation-caused reduction in NOL12 leads to apoptosis of retinal cells, we examined whether NOL12 interacts with ATR in the retinal cells. The ABC immunohistochemical staining showed that ATR was predominantly distributed in the cytoplasm of RGCs (Supplementary Fig. S2). Double immunofluorescent staining revealed that NOL12 was well colocalized with ATR in control WERI-Rb1 cells, predominantly in the nucleoli and to a lesser extent in the cytoplasm (Fig. 7A). In UV-exposed WERI-Rb1 cells, which exhibited lower levels of NOL12 and higher levels of ATR compared to control cells, colocalization of NOL12 and ATR was also detected, albeit to a lesser extent than in WERI-Rb1 cells without UV irradiation (Fig. 7A). In addition, co-immunoprecipitation assay displayed that a considerable amount of NOL12 was associated with ATR as well as with ATR’s substrate RPA32 in the WERI-Rb1 cells (Fig. 7B). Subsequent immunofluorescence double-labeling and co-immunoprecipitation analyses of NOL12 and ATR in the retinas of adult rats further demonstrated that NOL12 and ATR were colocalized in the cells of the retinal tissues, such as in RGCs, predominantly within the cytoplasm (Fig. 7C), and that NOL12 was notably associated with ATR in the retinal tissue of the adult rat (Fig. 7D).

**Fig. 8:**
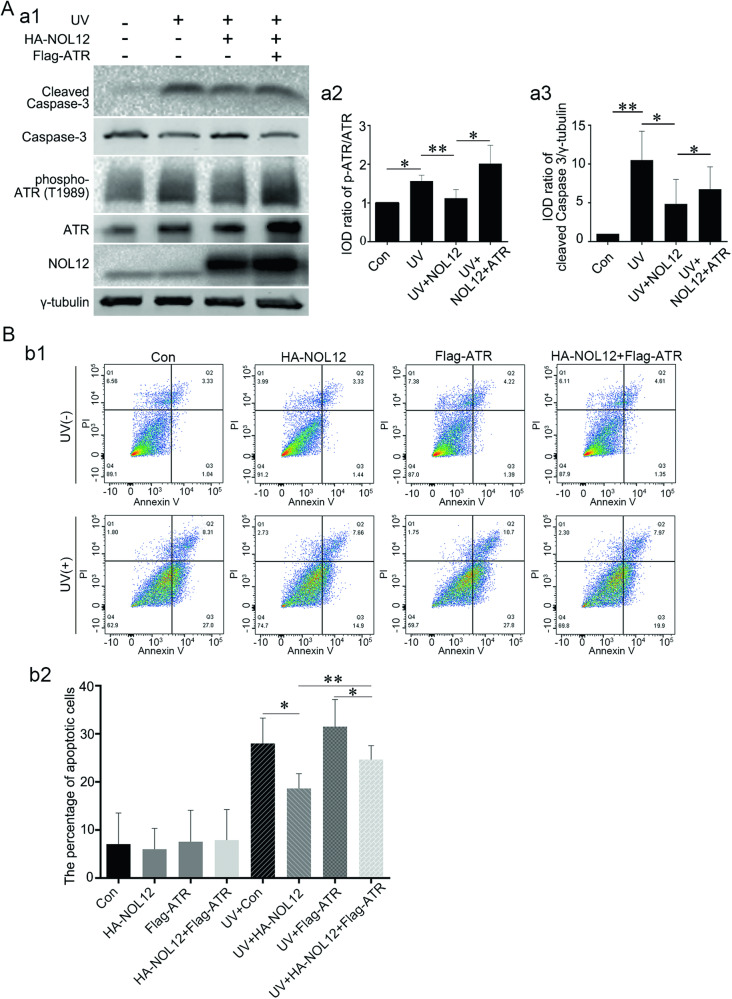
NOL12 inhibits the activation of ATR to protect against UV irradiation-induced apoptosis. **A** The expression of cleaved Caspase-3 and the activation of ATR were detected by Western blotting, showing that NOL12 overexpression inhibits the ATR activation and apoptosis induced by UV irradiation, while, ATR overexpression reverses the inhibitory role of NOL12 on the apoptosis. Rabbit polyclonal antibodies against NOL12 and ATR were utilized in Western blot. Representative Western blot bands are shown (a1), and the band intensities of p-ATR (a2) and cleaved Caspase-3 (a3) were analyzed. **B** Flow cytometric analysis was conducted to assess the impact of ATR overexpression on the inhibitory role of NOL12 in UV irradiation-induced apoptosis in WERI-Rb1 cells: Annexin V/PI flow cytometry was utilized to determine the apoptotic cells (b1), and the percentages of apoptotic cells underwent statistical analysis (b2). Data were expressed as mean ± SD. *n* = 3. *P < 0.05; **P < 0.01.

**Fig. 9:**
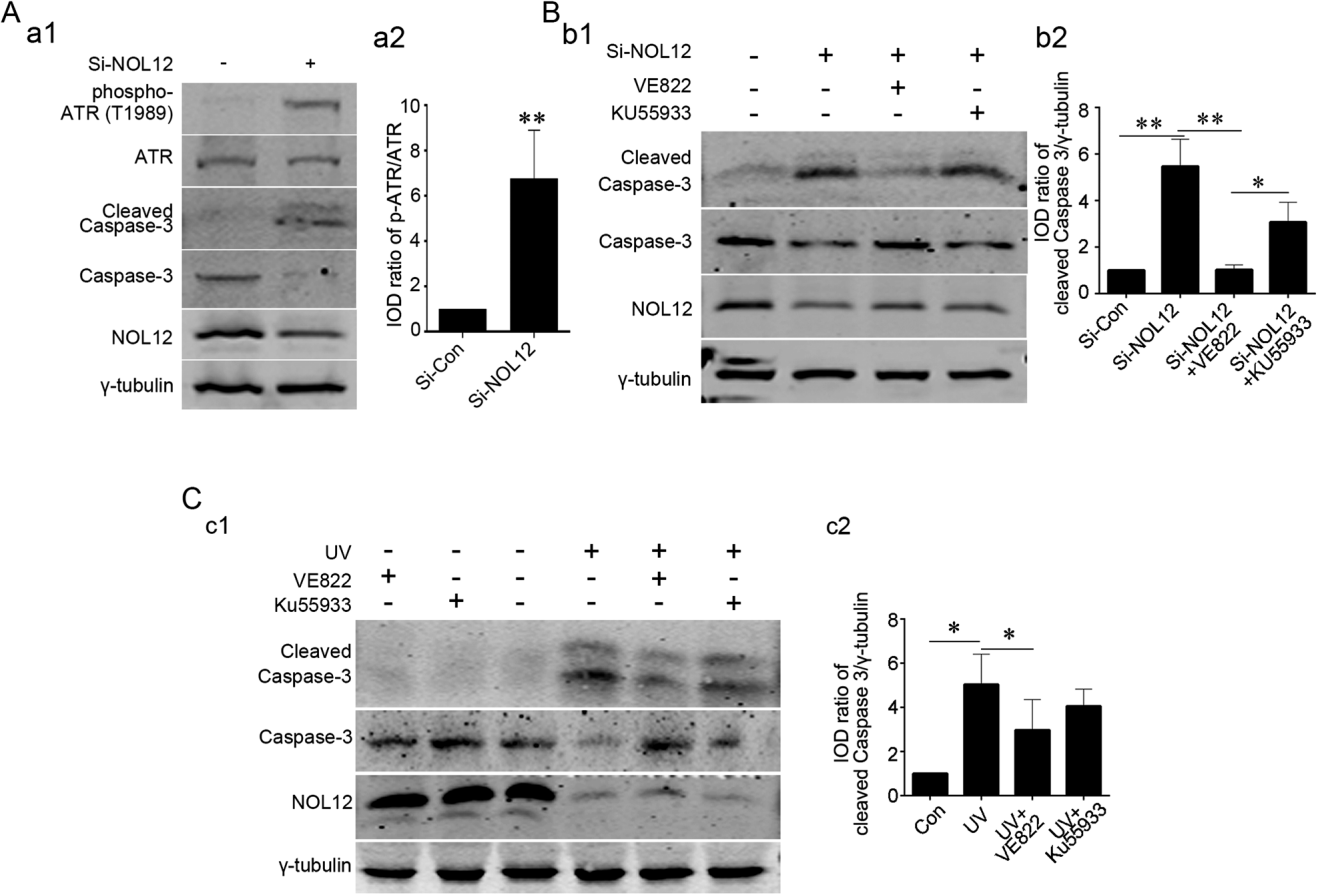
Inhibitor of ATR blocks the apoptosis induced by UV irradiation and NOL12 depletion. **A** The effect of silencing NOL12 expression with Si-NOL12 on the activation of ATR in WERI-Rb1 cells was analyzed by Western Blotting. **B**, **C** Detection of expression levels of cleaved Caspase-3 by Western blotting demonstrating that VE822 but not KU55933 inhibited apoptosis induced by NOL12 silencing (**B**) and UV irradiation (**C**). Rabbit polyclonal antibody against NOL12 and rabbit polyclonal antibody against ATR were utilized in Western blot. Representative Western blot bands were shown (a1, b1, and c1), and the band intensities were analyzed (a2, b2, and c2). Data were expressed as mean ± SD. *n* = 3. *P < 0.05, **P < 0.01.

Observing that NOL12 knockdown did not induce an increase in ATR expression, in contrast to the effect of ultraviolet radiation in WERI-Rb1 cells (Supplementary Fig. S3), we focused on analyzing the relationship between the UV-induced decrease in NOL12 levels and the alteration in ATR activity, and on exploring how the changes in ATR activity resulting from the decline of NOL12 expression correlate with apoptosis. As expected, irradiating WERI-Rb1 cells with UV caused not only significant upregulation of ATR level and downregulation of NOL12 level but also conspicuous enhancement of ATR activity, as indicated by the apparent increase in the level of phosphorylated ATR (ATR with phosphorylation at T1989, p-ATR) and in the ratio of p-ATR to ATR (Fig. 8A). Interestingly, overexpression of NOL12 obviously suppressed UV-induced enhancement of ATR activity, which was restored by ATR overexpression (Fig. 8A). Consistently, Western blotting and flow cytometric analysis on the relationship between ATR activity change related to NOL12 level changes and UV-induced apoptosis showed that the UV-induced activation of Caspase-3 was repressed by overexpressing NOL12, and this repression could be reversed, at least partially, by overexpression of ATR (Fig. 8A, B). Furthermore, it was observed that in WERI-Rb1 cells, silencing NOL12 not only led to the promotion of Caspase-3 cleavage but also caused a significant increase in ATR activity (Fig. 9A). Moreover, ATR inhibitor VE822, but not ATM inhibitor KU55933, attenuated UV-induced or Si-NOL12-induced Caspase-3 cleavage (Fig. 9B, C). Together, these results indicate that UV irradiation can trigger retina cell apoptosis by heightening ATR activity by promoting the degradation of NOL12.

## Discussion

NOL12 is a highly conserved mammalian nucleolar protein involved in regulating DNA/RNA metabolism and preserving nucleolus integrity [6, 7]. Its homolog in *Drosophila*, viriato, plays an important role in promoting eye disc growth and regulating photoreceptor differentiation during eye development [9, 10]. However, the expression and implications of NOL12 in the eyes of mammalian species remain unknown. In the present study, we demonstrate for the first time the expression of NOL12 protein in the adult rat retinal neurons and human retinal-derived WERI-Rb1 cell line, shedding light on its protective role against retinal damage caused by UV radiation.

NOL12 protein is predominantly localized to the nucleolus and nucleoplasm of a variety of cells, including HeLa, COS-7, HCT116, and primary fibroblasts [5–7, 29]. Similarly, in the WERI-Rb1 cells we examined, the majority of NOL12 was also found to be distributed in the nucleolus and nucleoplasm, though a low level of NOL12 was detected in the cytoplasm. However, unlike its distribution in these cell lines or primary cells, the majority of NOL12 in adult retinal neuronal cells was located in the cytoplasm, mirroring the expression pattern of NOL12 in the cytoplasm of the basal layer cells of stratified squamous epithelium in the human vagina [30]. The determination of this cytoplasmic localization indicates that NOL12 may have additional functions beyond the regulation of DNA/RNA metabolism and preservation of nucleolus integrity, tasks it accomplishes in the nucleus.

The proteins located in both cytoplasmic and nuclear subcellular compartments generally have the ability to shuttle between the nucleus and cytoplasm [31, 32]. We have previously found that amino acids 1–20 and 166–213 of NOL12 are the sequences responsible for its nuclear localization [30]. In addition, culturing HeLa cells with high concentrations of serum or treating HCT116 cells with actinomycin D, an apoptosis inducer, leads to a significant increase in NOL12 levels in the cytoplasmic compartment [6, 30]. In this study, we observed that in the retinal cells, the localization of NOL12 could shift from being predominantly nuclear during the embryonic stage to predominantly cytoplasmic in the postnatal and adult stages. Thus, it is suggested that NOL12 is a nuclear-cytoplasmic shuttling protein.

The role of NOL12 or its homolog viriato in protection and anti-apoptosis have been identified in the HCT116 cells and *Drosophila* eye disc cells, in which loss of NOL12 induces the trigger of apoptosis [6, 9, 10]. In the present work, we revealed that silencing NOL12 expression by siRNA suppresses cell viability, increases the apoptotic rate, and elevates the level of cleaved Caspase-3 in the WERI-Rb1 cells. Furthermore, it was found that overexpressing NOL12 effectively restrained UV radiation-induced rise in apoptotic rate and elevation of cleaved Caspase-3 level in WERI-Rb1 cells. Hence, It is indicated that maintaining a high level of NOL12 protein in the retina is imperative for protecting retinal neuronal cells against lesions, including apoptosis, induced by UV irradiation.

We detected decreased protein levels but increased mRNA levels of NOL12 in both WERI-Rb1 cells and rat retinal tissue in response to UV irradiation, possibly reflecting an increase in degradation rather than a decrease in expression of NOL12 when the retinal cells are exposed to UV. It is known that UV radiation activates several proteases, such as Caspases and calpains [33]. NOL12 contains several potential cleavage sites for Caspases and calpains (Supplementary Table S2). In fact, in HeLa cells treated with camptothecin, we have detected NOL12 protein degradation, which can be markedly inhibited by a pan-Caspase inhibitor Z-VAD-FMK [34]. Consistent with this finding, the UV irradiation-induced degradation of NOL12 in the present work can be effectively mitigated by Z-VAD-FMK treatment, but not by calpeptin (pan-calpain inhibitor) treatment, demonstrating that UV radiation degrades NOL12 protein in a Caspase-dependent manner. Collectively, these findings strongly suggest that Caspase pathway-dependent degradation of NOL2 protein by UV irradiation might be a new mechanism underlying retinal damage caused by UV stimulation. As several types of Caspases are activated by UV radiation [35], it is necessary to identify which type(s) of Caspase is/are activated by UV stimulation to produce NOL12 degradation.

It is known that high doses of UV irradiation cause severe DNA damage and cell apoptosis in the retina [21, 24, 25]. Severe DNA damage leads to the activation of ATR and subsequent cellular lesions, including apoptosis [19, 36, 37]. Consistently, our study shows a high dose of UV irradiation induces obvious apoptosis as well as enhancement of ATR activity in the WERI-Rb1 cells, as evidenced by elevated phosphorylation levels of ATR. To confirm the function of ATR in the retina, ATR distribution was assessed using ABC staining, demonstrating a predominantly cytoplasmic expression, consistent with the cytoplasmic expression in A549 cells [38]. Moreover, NOL12 is found to colocalize and interact with ATR in the nuclei, as well as in the cytoplasm, of both WERI-Rb1 cells and the cells of retinal tissue, suggesting that NOL12 may play its anti-apoptotic role via ATR. Supportively, overexpression of NOL12 restrains the rise in ATR activity and augmentation of apoptosis in UV-irradiated WERI-Rb1 cells, which can be counteracted by overexpression of ATR. In addition, silencing NOL12 increases the activity of ATR in the apoptotic WERI-Rb1 cells, and apoptosis in the WERI-Rb1 cells produced by silencing NOL12 is significantly abated by the administration of ATR inhibitor. Accordingly, it is indicated that UV radiation-elicited DNA damage over-activates ATR, triggering retinal apoptotic injury, and that NOL12, as a protective protein, alleviates the apoptotic lesion of retinal cells induced by UV irradiation by inhibiting the overactivation of ATR. Thus, the UV-induced overactivation of ATR might be correlated with a reduction in the inhibitory effect of NOL12 on ATR activation, a reduction possibly resulting from the elevated degradation of NOL12 following UV exposure.

The ATR activation resulted from severe DNA damage initiates the downstream Chk2/p38-MAPK signaling pathway, a process distinct from the Chk1/SMARCAL1 pathway engaged by ATR response to low-level DNA damage [36, 37, 39, 40]. Further explorations are needed to determine if the ATR activation caused by a high dose of UV irradiation on the WERI-Rb1 cells leads to upregulation of the Chk2/p38-MAPK signaling pathway, if enhanced expression of NOL12 weakens the impact of high-dose UV irradiation on the engagement of this signaling pathway, and if the modulatory effects of NOL12 on the ATR-Chk2/p38-MAPK pathway under high-dose UV irradiation are observable in animal retinas. Such investigations would help to further substantiate the important role of NOL12 in protecting the retina from UV-induced lesions by restraining the overactivation of ATR. Considering that diseases such as ATR-related Seckel syndrome and Werner syndrome are associated with the abnormal activation of ATR and the downstream p38-MAPK singling pathway [41, 42], it is therefore suggested that NOL12 may serve as a potential therapeutic target for diseases related to abnormal ATR activation.

## Materials and methods

### Plasmids and small interfering RNAs

Plasmid pCMV-HA was a kind gift from Prof. Yanhong Liao (Huazhong University of Science and Technology, China). Plasmid pCMV-HA-NOL12 was constructed by in-frame insertion of human NOL12 *cDNA*, which was obtained from HEK293T cells, into the pCMV-HA expression vector. Plasmids pCMV-flag-ATR and pCMV-flag were purchased from Addgene (MA, USA). Small interfering RNA (siRNA) targeting *NOL12* (si-NOL12) and a scrambled control siRNA encoding a nonspecific sequence (si-Con) were synthesized by Guangzhou Ribo Biotechnology (Guangzhou, China). The gene sequences used to design the siRNAs are AGAAGCGAGATGGTGACGA for si-NOL12 and TTCTCCGAACGTGTCACGT for si-Con.

### Reagents

We purchased a range of antibodies and reagents for our research. From Proteintech Group (IL, USA), we acquired rabbit polyclonal antibodies against NOL12 (15456-1-AP), ATR (19787-1-AP), and MAP2(17490-1-AP), as well as mouse monoclonal antibodies against Caspase-3 (66470-2-Ig), fibrillarin (66985-1-Ig). From Santa Cruz Biotechnology (Texas, USA), we obtained mouse monoclonal antibodies targeting NOL12 (sc-374257), ATR (sc-515173), BRN3B (sc-515173), and GAP43(sc-17790). Sigma (MO, USA) supplied us with the mouse monoclonal antibody against γ-tubulin (T6557), while Cell Signaling Technology (MA, USA) provided the rabbit monoclonal antibody against cleaved Caspase-3 (9661). Abcam (MA, USA) was the source of our rabbit monoclonal antibody against phospho-ATR (T1989, ab289363) and the mouse monoclonal antibody against RPA32 (ab2175). From Jackson ImmunoResearch (PA, USA), we procured various conjugated secondary antibodies, including biotinylated goat anti-rabbit/mouse IgG, RRX-conjugated goat anti-rabbit/mouse IgG, FITC-conjugated goat anti-rabbit/mouse IgG. LI-COR technology (NE, USA) supplied us with IRDye 800CW labeled goat anti-rabbit IgG and IRDye 680CW labeled goat anti-mouse IgG.

We also used a range of other reagents from various suppliers for different purposes in our experiments, including the NOL12 fusion protein (Ag7738) from Proteintech Group (IL, USA); streptavidin–biotin-peroxidase complex, Hoechst 33258, DAB, His-tag affinity magnetic beads, PMSF, and a protease inhibitor cocktail from Sigma-Aldrich (MO, USA); RPMI 1640 medium, Lipofectamine 2000^TM^, Pierce BCA Protein Assay Kit, and TRIzol reagent from Thermo Fisher Scientific (MA, USA); Protein A + G beads from Beyotime (Shanghai, China); ATR inhibitor VE822, ATM inhibitor ku55933, Caspase inhibitor Z-VAD-FMK, and calpain inhibitor calpeptin from MedChemExpress (NJ, USA); Alamar Blue kit, TUNEL staining kit, and SYBR Green PCR Mix Kit from Yeasen Biotech (Shanghai, China); Annexin V/PI Apoptosis Detection Kit from KeyGEN (Jiangsu, China); Taqman Universal PCR mix from Tiangen Biotech (Beijing, Chian); GoScript™ reverse transcription kit from Promega (WI, USA).

### Animals

Sprague Dawley (SD) rats, including non-pregnant rats aged 6–8 weeks, pregnant rats on day 13.5 of pregnancy, and newborn SD rats at 1 day postpartum, were obtained from the Experimental Animal Center of Tongji Medical College, Huazhong University of Science and Technology (Wuhan, China). They were housed in a temperature and humidity-controlled, specific pathogen-free environment. All animal experiments were conducted in accordance with the guidelines for animal research and approved by the Animal Ethics Committee of Tongji Medical College, Huazhong University of Science and Technology.

### Cell culture and transfection

The human retinoblastoma cell line WERI-Rb1, authenticated by STR profiling and tested for Mycoplasma contamination prior to use, was purchased from the ICells Company (Shanghai, China). It was cultured in RPMI 1640 medium containing 10% fetal bovine serum, 100 U/mL penicillin, and 100 µg/mL streptomycin at 37 °C in a humidified 5% CO_2_ incubator. For transfection, the cells were plated and grown until they reached 40–50% confluence, and then transfected with corresponding plasmids or small interfering RNAs (si-NOL12 or si-Con) using Lipofectamine 2000^TM^ reagent according to the manufacturer’s protocol.

### Inhibitors treatment of cells

WERI-Rb1 cells were treated with ATR inhibitor VE822 (1 μM), ATM serine/threonine kinase (ATM) inhibitor KU55933 (1 μM), Caspase inhibitor Z-VAD-FMK (10 μM), or calpain inhibitor Calpeptin (10 μM), each for 1 h prior to transfection or the UV irradiation. These doses were selected based on dose-response experiments, which demonstrated that at these concentarations, the kinases were effectively inhibited without initiating an apoptotic response.

### UV irradiation

For UV irradiation treatment of the rat eyes, the right eye of each anesthetized rat was irradiated with UV light (UV emitter, Leinan, Wuhan, China) at 400 J/m^2^, and the left eye of the same rat, which were not irradiated, served as control [43]. Considering the exploratory nature of the animal experiments, a minimal sample size of 6 animals was initially estimated using the resource equation method for sample size calculation. As our UV irradiation experiment employed an autologous control within the same individual, no randomization was used in animal group allocation. To ensure blinding, the model development and subsequent testing phases were conducted by different researchers. There were no exclusion criteria. For the UV irradiation treatment of WERI-Rb1 cells, the cells were cultured on poly-l-lysine (PLL)–coated glass chamber slides and irradiated with UV light (CL1000, UVP, Jena, Germany) at 50 J/m^2^. The analysts conducting the evaluations were also blinded to the group assignments of the samples.

### Immunohistochemistry

Immunohistochemistry staining was performed as previously described [44]. For immunohistochemical detection of NOL12 and ATR in the retina with the ABC method, the rat eyes were collected, fixed with 4% paraformaldehyde, embedded in paraffin, and serially sectioned at a thickness of 8 µm. Following this, antigen retrieval was performed using a sodium citrate buffer at pH 6.0. The sections were then permeabilized with 3% Triton X-100 and treated with 3% H_2_O_2_. This was succeeded by blocking with 3% bovine serum albumin (BSA) to prevent nonspecific binding. The sections were then incubated with the primary antibody against NOL12 or ATR both at a dilution of 1:200 overnight at 4 °C. Subsequently, they were incubated with biotinylated goat anti-rabbit/mouse IgG and streptavidin-biotin-peroxidase complex, both at a dilution of 1:200. Peroxidase activity, indicative of NOL12 or ATR presence, was visualized using a liquid DAB substrate chromogen system. After these steps, the sections underwent dehydration, clearing, and were cover-slipped. Finally, detailed examination and imaging of the sections are carried out using a Nikon DXM1200 CCD camera (Tokyo, Japan), providing critical insights into the distribution and localization of NOL12 or ATR in the retinal tissue. Additionally, the specificity of the anti-NOL12 antibody used in this process is rigorously verified through an absorption experiment. In this validation step, the anti-NOL12 antibody is preabsorbed with an excess of 6×His tagged NOL12 fusion protein, ensuring its selective binding to the target NOL12 in the samples. The specific distribution of NOL12 was also confirmed by rabbit- and mouse-derived anti-NOL12 antibodies.

For single immunofluorescence staining of NOL12 in retina and WERI-Rb1 cells, paraffin-embedded sections and WERI-Rb1 cells fixed with 4% paraformaldehyde were permeabilized and blocked, and incubated sequentially with anti-NOL12 antibody (1:200) and RRX-conjugated goat anti-rabbit IgG (1:200), followed by staining with Hoechst 33258 at a concentration of 1 µg/mL, targeting the nuclei for visualization. Imaging of the stained samples was then performed using an Olympus FV1000 laser-scanning confocal microscope (Tokyo, Japan), to capture pictures.

In the process of immunofluorescence double staining, retina sections, and WERI-Rb1 cells were first subjected to a series of pretreatment steps as previously outlined. Following this, the samples were incubated with various antibody combinations at 4 °C overnight. These combinations included mouse anti-NOL12 at a 1:100 dilution paired with rabbit anti-MAP2 at 1:200, and rabbit anti-NOL12 at 1:200 combined with mouse anti-GAP43 at 1:200. Other pairings used were rabbit anti-NOL12 at 1:200 with mouse anti-BRN3B at 1:200, rabbit anti-NOL12 at 1:200 with mouse anti-ATR at 1:100, and rabbit anti-NOL12 at 1:200 with mouse anti-fibrillarin at 1:200. Subsequent to this primary antibody incubation, the samples underwent a further incubation phase with a mixture of RRX-conjugated goat anti-rabbit/mouse IgG and FITC-conjugated goat anti-mouse/rabbit IgG, both at a dilution of 1:200. Following antibody incubation, the nuclei in the samples were stained with Hoechst 33258 at a concentration of 1 µg/mL for visualization. Finally, the slides were mounted using 30% glycerol in PBS, and images were acquired using the laser-scanning confocal microscope.

### Alamar Blue assay

Cell viability was assessed using the Alamar Blue assay, following the manufacturer’s protocol. Initially, cells, at a cell density of 1 × 10^5^ per well, were plated in 96-well plates and subsequently transfected with the appropriate plasmids and siRNAs. After a period of 48 h, the Alamar Blue reagent was added to each well. The cells treated with the reagent were incubated for 4 h at a temperature of 37 °C. Following this incubation, cellular fluorescence, indicative of cell viability, was measured at a wavelength of 590 nm, utilizing a microplate reader (Enspire, PerkinElmer, OH, USA).

### Western blotting

The tissues and the WERI-Rb1 cells were collected and homogenized using a lysis buffer composed of 50 mM NaCl, 50 mM Tris-HCl, 1 mM EDTA, pH 7.4, 1% Triton X-100, and a combination of protease and phosphatase inhibitors. Following homogenization, protein concentrations were determined using the Pierce BCA Protein Assay Kit. The protein samples were then resolved on SDS polyacrylamide gels and subsequently transferred onto nitrocellulose membranes. After blocking with 5% fat-free milk in PBS, membranes were incubated overnight at 4 °C with a variety of primary antibodies. These antibodies included rabbit anti-NOL12, rabbit anti-cleaved Caspase-3, mouse anti-γ-tubulin, rabbit anti-ATR, rabbit anti-ATR (phospho T1989), mouse anti-RPA32, and mouse anti-Caspase-3, all diluted at specific concentrations (1:1000 for most, except γ-tubulin at 1:10000). Post-primary antibody incubation, the membranes were washed and then incubated for 1 h at room temperature with a mixture of IRDye 800CW labeled goat anti-rabbit IgG and IRDye 680CW labeled goat anti-mouse IgG. The final step involved capturing images and measuring the integrated optical densities (IOD) of the protein bands using Odyssey® CLx Imaging System (LI-COR, NE, USA).

### Semi-quantitative RT-PCR and quantitative RT-PCR analysis

RNA isolation was performed using TRIzol reagent, followed by reverse transcription with random hexamers and the GoScript™ reverse transcriptase following the manufacturer’s instructions. For semi-quantitative RT-PCR analysis, the products of the reverse transcription were amplified via PCR using Taqman Universal PCR mix in a PCR system (Eppendorf, Westbury, USA). The amplified PCR products were then separated by agarose gel electrophoresis and visualized after staining with ethidium bromide using the ChemiDoc Imaging System (Bio-Rad Laboratories, CA, USA). To quantify specific mRNA expression levels, β-actin mRNA served as the internal control. The intensity of each gel band was measured using the ImageLab software (Bio-Rad Laboratories, CA, USA). The ratio of the specific mRNA to β-actin mRNA of each sample (relative intensities) was calculated to compare the expression levels of the specific mRNAs across different samples. For quantitative real-time RT-PCR (qRT-PCR) analysis, a SYBR Green PCR Mix Kit was employed for qPCR in the StepOnePlus real-time PCR system (Applied Biosystems, CA, USA). The qRT-PCR data were analyzed by the ∆∆Ct method, with each specific mRNA expression level normalized against β-actin mRNA expression. The primer sequences used for both semi-quantitative RT-PCR and qRT-PCR were listed in Supplementary Table S1.

### Apoptosis assay

Apoptosis was examined by Annexin V/PI flow cytometry and TUNEL assay. In the flow cytometry approach, cells were collected, washed with PBS, and subsequently stained with a mixture of Annexin V and PI reagents in a binding buffer at room temperature for 15 min. The stained cells were subsequently analyzed using an LSR II Flow Cytometer (Becton Dickinson, MA, USA). The resulting data were processed and interpreted using FlowJo Software V10 (FlowJo, LLC, USA). For the TUNEL assay, a TUNEL staining kit was utilized. Briefly, the frozen retinal tissue sections and WERI-Rb1 cells fixed with 4% paraformaldehyde were permeabilized with 3% Triton X-100 for 1 h, and then stained with Alexa fluor 488-12-dUTP in a dark environment at 37 °C for 1 h. Following this staining, counterstaining was performed with Hoechst 33258, and the samples were mounted with glycerol in PBS. The final analysis was conducted under a laser-scanning confocal microscope. The rate of apoptosis in the samples was quantified by counting the total number of TUNEL-positive cells relative to the total cell count.

### Co-immunoprecipitation assay

Cells or retinas were harvested and lysed with immunoprecipitation lysis buffer composed of 50 mM Tris-HCl, 50 mM NaCl, 1 mM MgCl_2_, 1 mM EDTA, 1% Triton X, 10% glycerol, pH 7.4, supplemented with protease and phosphatase inhibitors. The lysates were then subjected to pre-clearing with Protein A + G beads. Following this, they were incubated with specific antibodies, either rabbit anti-NOL12 at a 1:100 dilution or rabbit anti-ATR at a 1:50 dilution, at 4 °C overnight. This was followed by a further incubation with A + G beads at 4 °C for 2 h. Subsequently, the beads were washed using a wash buffer consisting of 50 mM Tris-HCl, 150 mM NaCl, 1 mM EDTA, and 0.1% NP-40, pH 7.4. The proteins bound to the beads were then eluted with a 2× SDS sample buffer. Finally, these bead-bound proteins were separated by SDS-PAGE and detected by immunoblotting using the indicated antibodies.

### Statistical analysis

Statistical analyses of the data were conducted using SPSS software V 21.0 (IBM, IL, USA). The qualitative data and cell images presented are representative of a minimum of three independent experiments. All quantitative data were expressed as the mean ± standard deviation. To evaluate differences between groups, statistical tests, including the Student’s *t*-test and ANOVA test were employed. A *P*-value of less than 0.05 was considered statistically significant.

### Supplementary information

SUPPLEMENTAL MATERIAL

Original gel

## Data Availability

All data on which the conclusions of the paper rely has been provided in the paper.
