# Supplementary material for: Protein expression of nucleolar protein 12 in the retina and its implication in protection of retina from UV irradiation damage
Source: Cell Death Discov. 2024 Mar 11;10:130. doi: 10.1038/s41420-024-01902-x (PMC10928217; doi:10.1038/s41420-024-01902-x)
Supplement: Supplementary file 1 — SUPPLEMENTAL MATERIAL [file 41420_2024_1902_MOESM1_ESM.pdf]

Supplementary table S1 Primer Sequences used in RT-PCT and qRT-PCR.

Gene	Host	Sequences 5′→3′	Length (bp)
NOL12	Human	F: GAGGCGTCATCCACGGAGAAACC	140
		R: CGTCGGGGATGTTTCCTCTTGACC	
NOL12	Rat	F: CCTGAAGATGCTGGCAGAAAGAG	140
		R: GGTCCAGGTCACTGACGGTGGT	
Actin	Human	F: AGAAGAGCTACGAGCTGCCTGACG	125
		R: GGTAGTTTCGTGGATGCCACAGGAC	
Actin	Rat	F: CCGATGCCCCGAGGCTCTCTTCCAG	118
		R: ATAGAGGTCTTTACGGATGTCAACG	
ATR	human	F: AGGACTACCAGCACAACCAGC	138
		R: AAGGGAAATAGTGTCTTTATCAGC	

Supplementary table S2 The predicted protease cleavage sites of NOL12.

Predicted protease	Position of NOL12	Site of NOL12	Similarity maxsite	Frequency score	Similarity score
Caspase	83 to 88	DELDRL	NEIDRL	5.19	76.67
	80 to 85	EEADEL	QEADEA	17.74	72.41
	111 to 116	SDLDLS	SELDAS	0.26	70.37
	183 to 188	RAQDSK	REQDSE	0.02	66.67
	20 to 25	LSFDEE	IQFDSE	0.19	63.33
	9 to 14	RDGDDR	EDEDDK	0.05	54.55
	96 to 101	VQYDHP	VEVDAP	0.08	53.33
	10 to 15	DGDDRR	DEDDKT	0.21	51.52
	109 to 114	TISDLD	TLTDSS	0.17	42.86
Calpain	162 to 167	SSLTAS	SALTAS	60.00	88.00
	83 to 88	DELDRL	DQLDAI	2.62	65.52
	51 to 56	QRLKEE	RRLLQE	3.25	53.57
	154 to 159	DPLLSQ	EKLKSQ	11.02	42.86
	134 to 139	SEEEAS	AEIEAI	8.89	38.46
	111 to 116	SDLDLS	RGLSLS	5.77	37.04

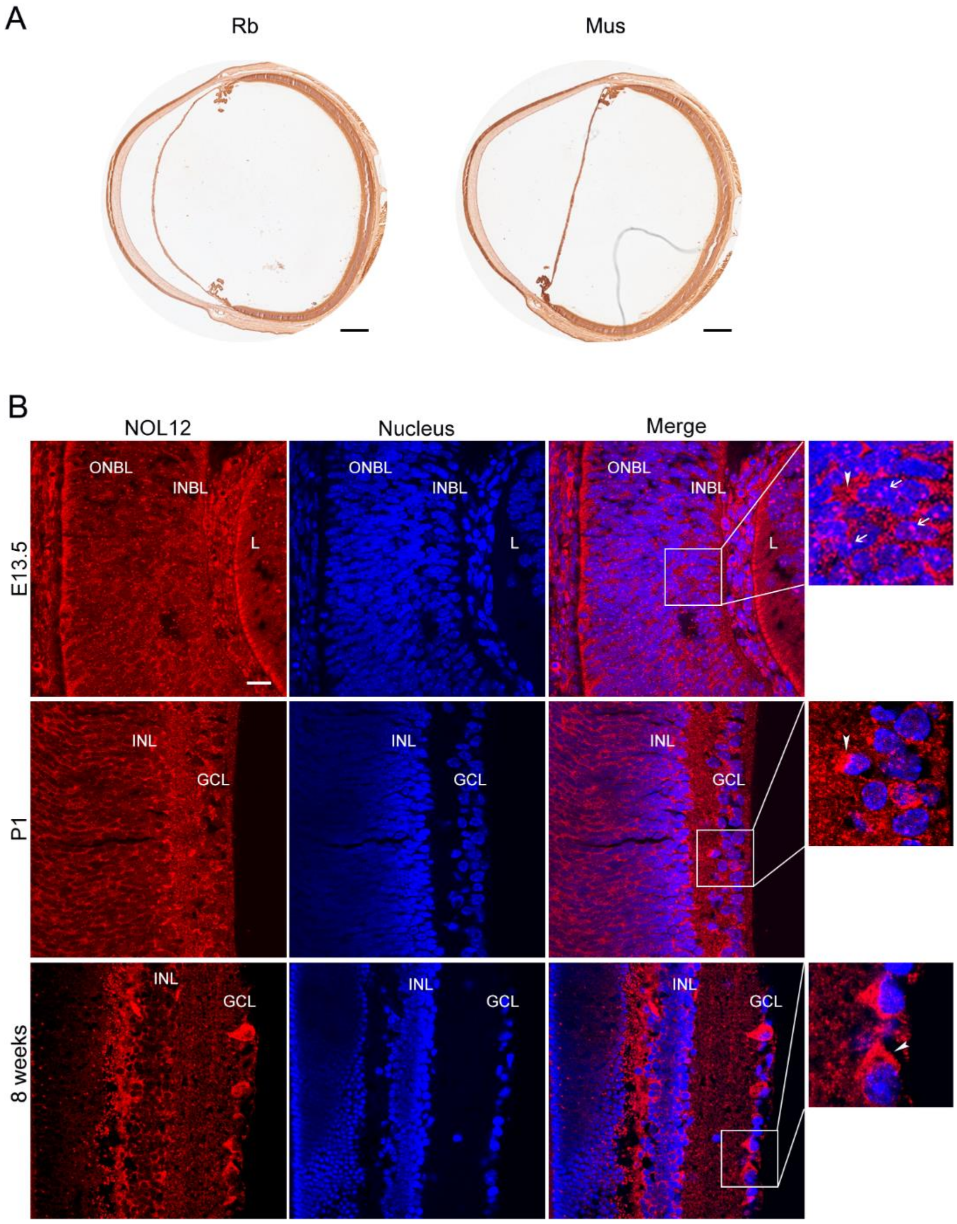

Supplementary Fig. S1 The differential expression patterns of NOL12 in developing rat retinas. A Immunohistochemical examination of NOL12 distribution in the retinas of adult rats, showing that rabbit-derived and mouse-derived NOL12 antibodies have the same immunoreactivity. Rabbit polyclonal antibody against NOL12 and mouse monoclonal antibody against NOL12 were used in ABC staining. **B** Immunofluorescence staining for analyzing subcellular localization of NOL12 in

the developing retinal cells, showing that in E13.5, NOL12 was expressed in the nucleus (**arrows**) and cytoplasm (**arrowheads**) of INBL, while in P1 and adult rats, NOL12 was expressed in the cytoplasm (**arrowheads**) of GCL. Rabbit polyclonal antibody against NOL12 was utilized in immunofluorescence staining. INBL, inner neuroblastic layer; ONBL, outer neuroblastic layer; L, lens; INL, inner nuclear layer; GCL, ganglion cell layer. Scale bars: 500 μm in A, 20 μm in B.

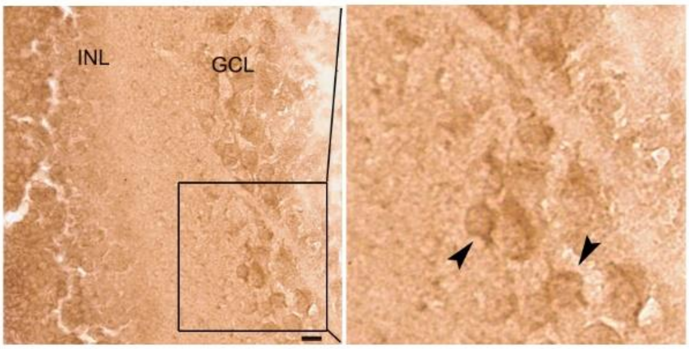

Supplementary Fig. S2 ATR is expressed in the cytoplasm of retinal cells.

Expression analysis of ATR in adult rat eyes was conducted using the ABC staining. Arrowheads indicate the cytoplasmic expression of ATR. ONL, outer nuclear layer; INL, inner nuclear layer; GCL, ganglion cell layer. Scale bars: 10 μm.

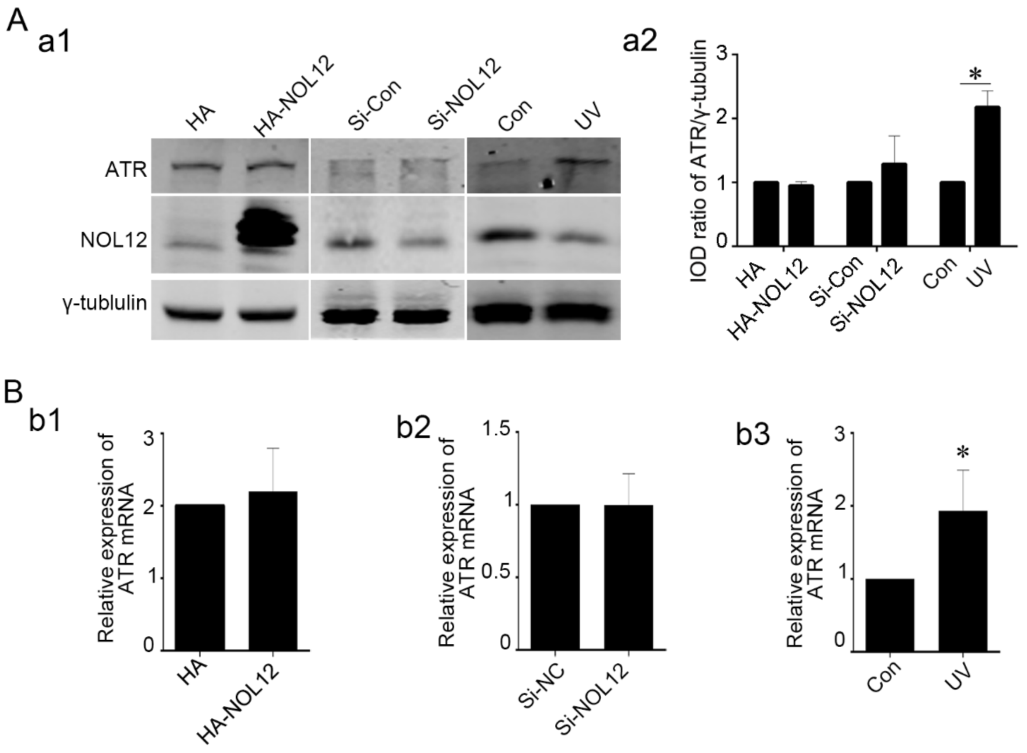

Supplementary Fig. S3. NOL12 does not affect the expression of ATR in WERI-Rb1 cells. **A** The effects of NOL12 overexpression, NOL12 silencing and UV irradiation on the expression of ATR protein levels were detected by Western blotting. Rabbit polyclonal antibody against NOL12 and rabbit polyclonal antibody against ATR were utilized in Western blot. The representative Western blot bands were shown (**a1**) and the band intensities were analyzed (**a2**). **B** The effects of NOL12 overexpression, NOL12 silencing and UV irradiation on the expression of ATR mRNA were measured using qRT-PCR. Data are expressed as mean±SD. n=3. * $P<0.05$.
