# Supplementary figures and images for: Protein expression of nucleolar protein 12 in the retina and its implication in protection of retina from UV irradiation damage
Source: Cell Death Discov. 2024 Mar 11;10:130. doi: 10.1038/s41420-024-01902-x (PMC10928217; doi:10.1038/s41420-024-01902-x)

Figure 1

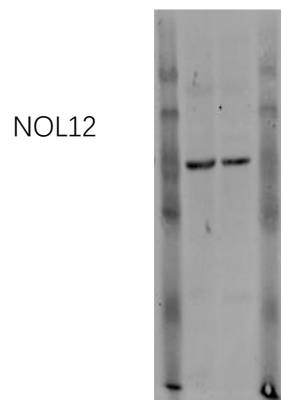

Figure 3

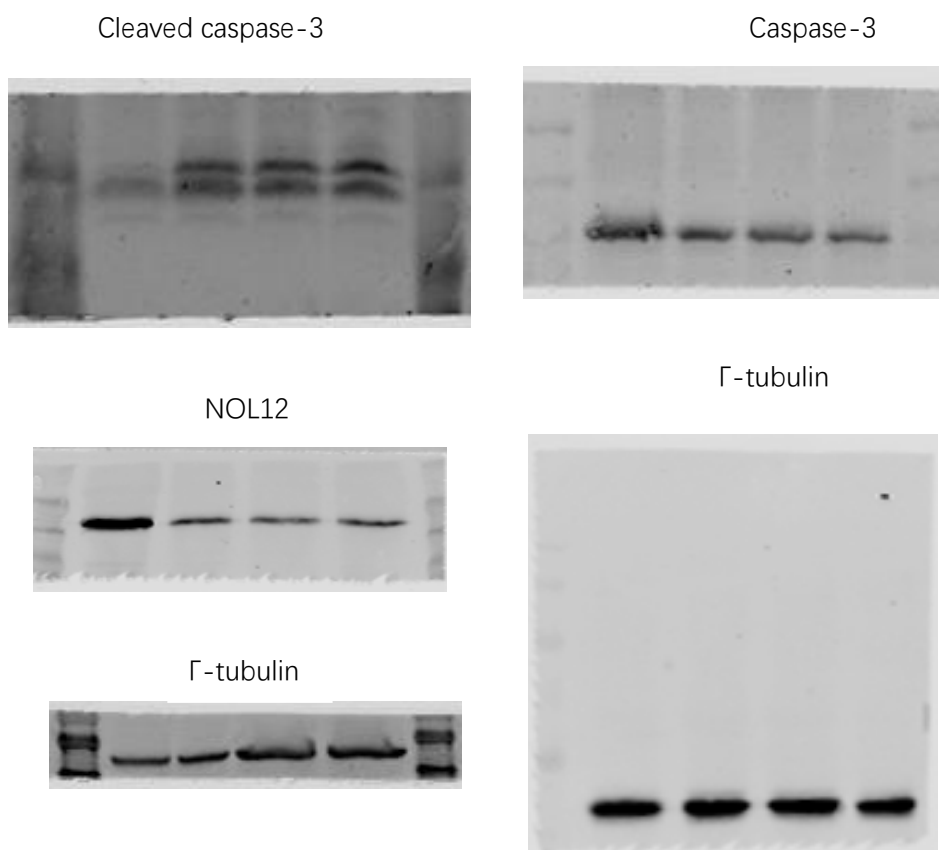

Figure 4

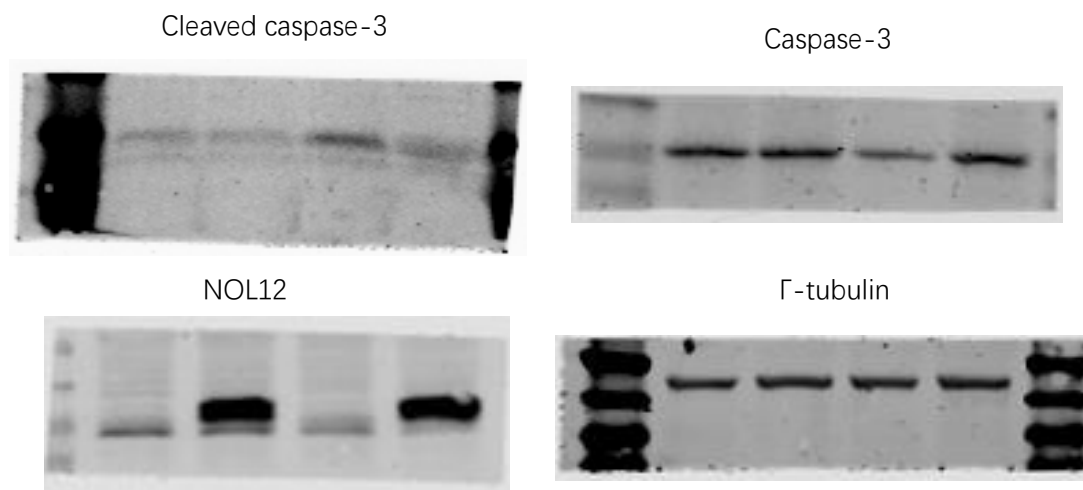

Figure 5

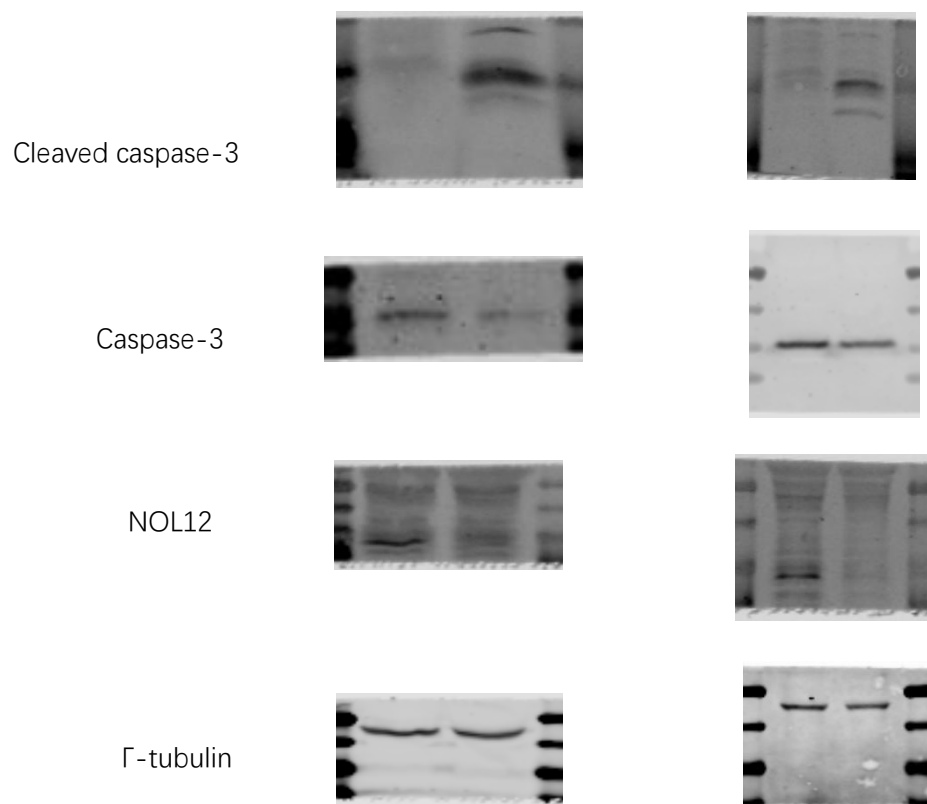

Figure 6

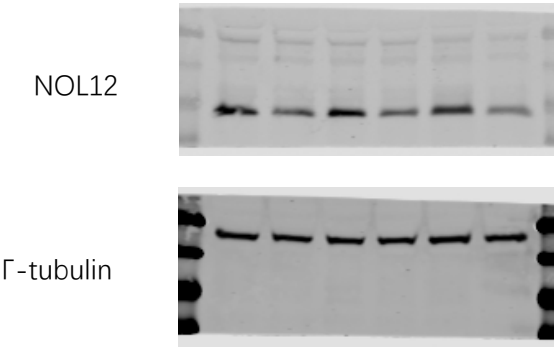

Figure 7

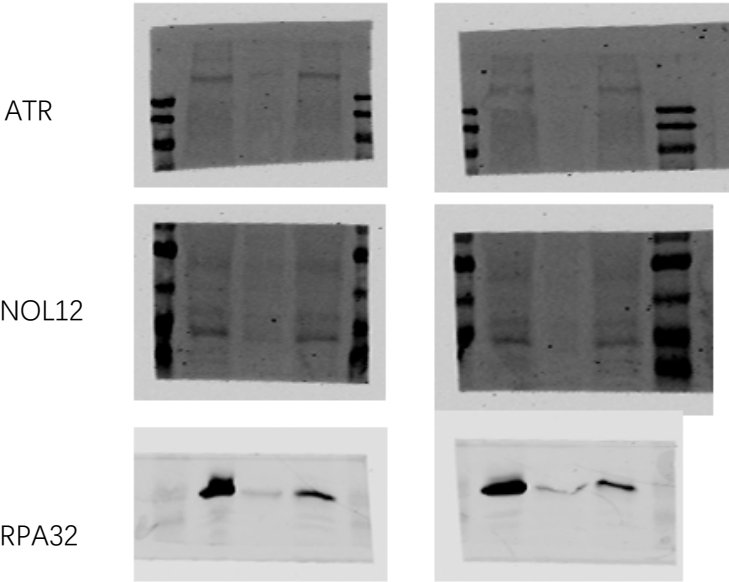

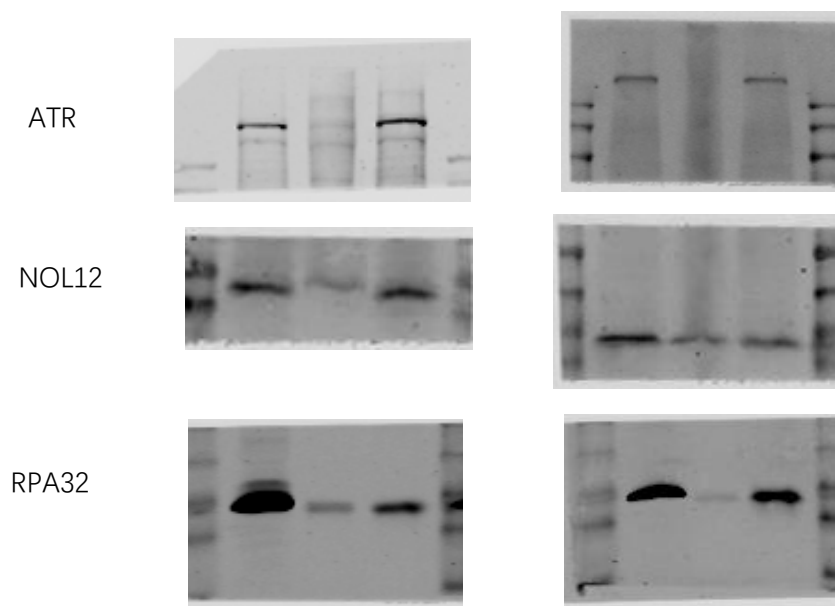

Figure 8

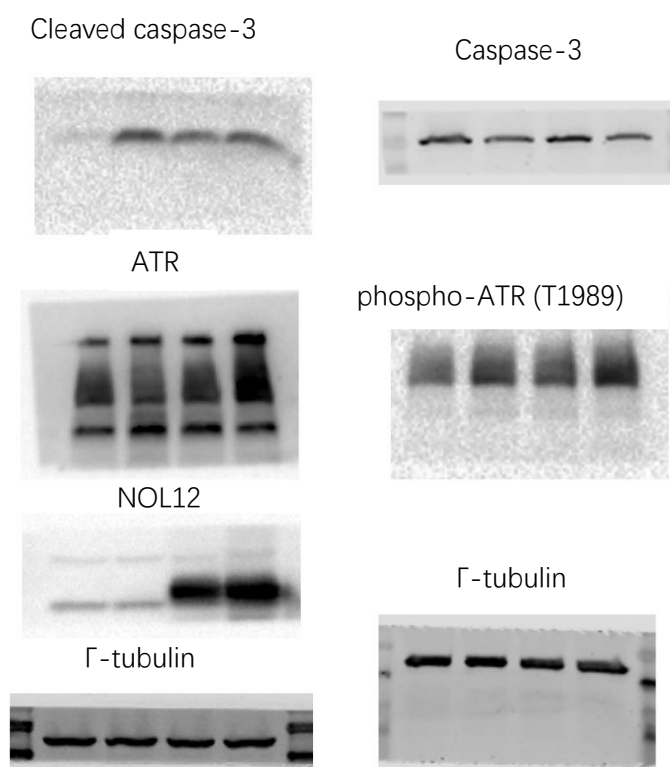

Figure 9

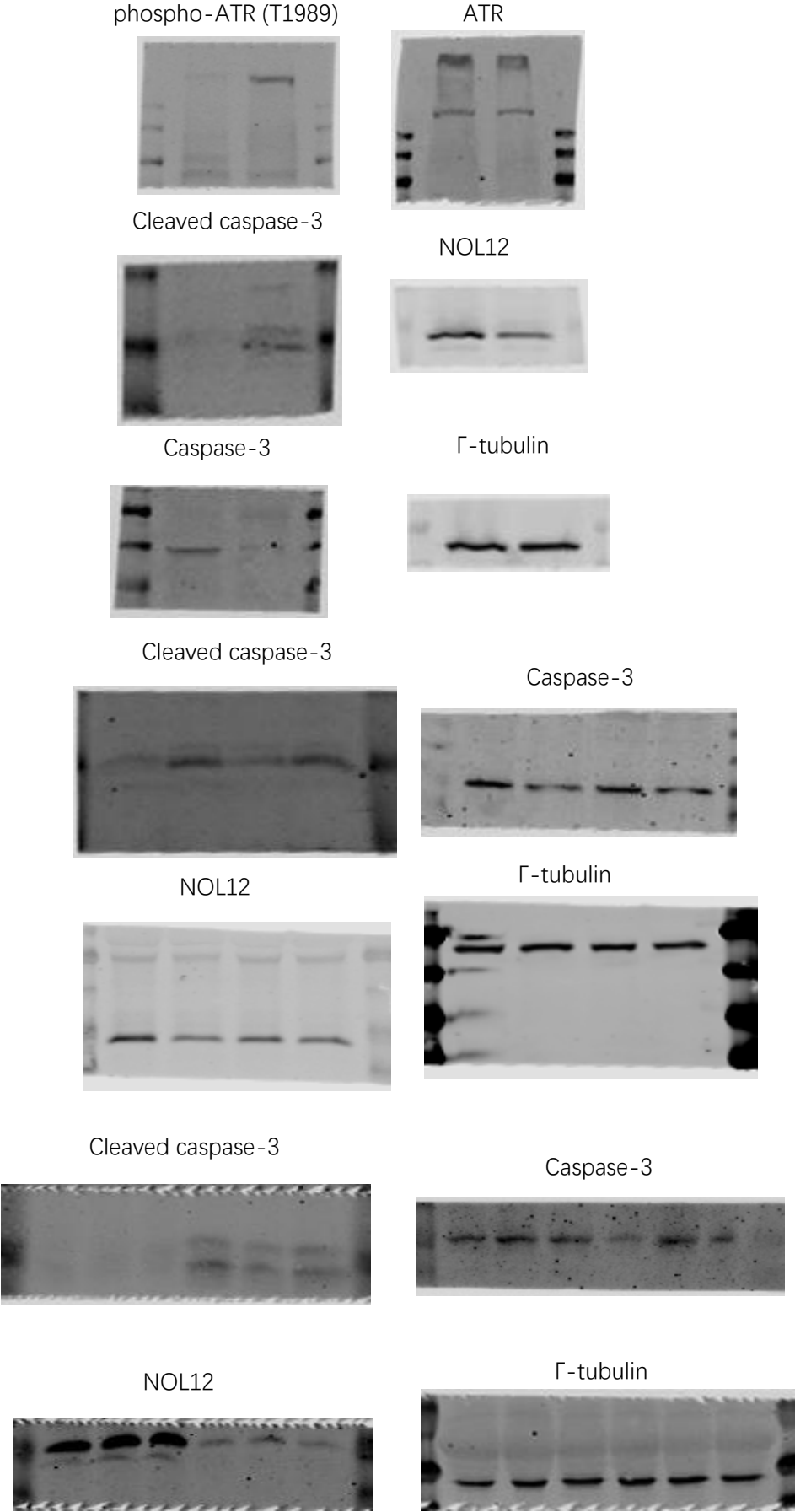

Supplementary Figure S3

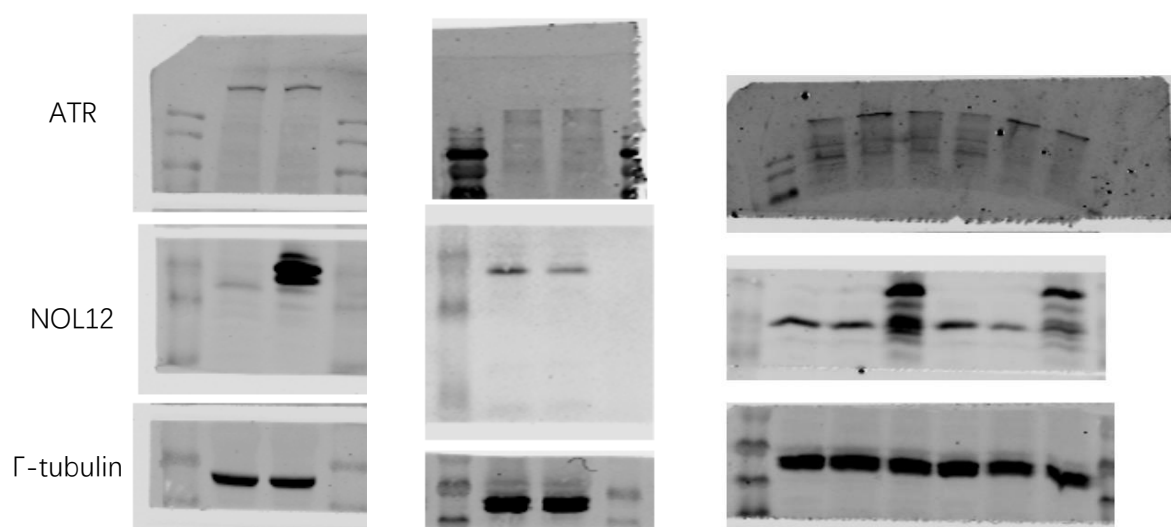

Supplement: Supplementary file 2 — Original gel [file 41420_2024_1902_MOESM2_ESM.pdf]
